# Effect of Hyperhomocysteinemia on Clinical Outcome and Hemorrhagic Transformation After Thrombolysis in Ischemic Stroke Patients

**DOI:** 10.3389/fneur.2019.00592

**Published:** 2019-06-04

**Authors:** Yun Luo, Hang Jin, Zhen-Ni Guo, Peng Zhang, Li-Yue Zhang, Jie Chen, Yao Yu, Yan Wang, Jie Liu, Qian-Yan He, Xin Sun, Yi Yang

**Affiliations:** ^1^Department of Neurology, Stroke Center, The First Hospital of Jilin University, Chang Chun, China; ^2^Department of Neurology, Neuroscience Center, The First Hospital of Jilin University, Chang Chun, China

**Keywords:** hyperhomocysteinemia, ischemic stroke, recombinant tissue plasminogen activator, intravenous thrombolysis, clinical outcome, hemorrhagic transformation, symptomatic intracerebral hemorrhage

## Abstract

**Background and Purpose:** Hyperhomocysteinemia (Hhcy) is a well-known risk factor for ischemic stroke. However, the role of Hhcy in the clinical outcome of ischemic stroke has not been fully elucidated. In addition, previous studies have found that Hhcy was implicated in the disruption of the blood-brain barrier, which may increase the risk of hemorrhagic transformation (HT) after thrombolysis. Thus, the aim of this study was to investigate the effect of Hhcy on the clinical outcome and HT after thrombolysis in ischemic stroke patients.

**Methods:** Patients who were diagnosed with ischemic stroke and received intravenous thrombolytic therapy between January 2016 and September 2018 were included in this study. Multivariate logistic regression analysis was used to assess the association between Hhcy, clinical outcome, and HT after thrombolysis. Furthermore, the potential interaction between Hhcy and hypertension on the clinical outcome and HT after thrombolysis was also assessed.

**Results:** Of 568 patients, 455 (80.1%) had Hhcy, 66 (11.6%) had HT, and 219 (38.6%) had poor outcome. Patients with Hhcy had a higher incidence of poor outcome than the patients with non-Hhcy (40.9 vs. 29.2%, *p* = 0.022). However, there was no significant difference in the incidence of HT (11.9 vs. 10.6%, *p* = 0.711) between patients with Hhcy and non-Hhcy. After adjustment for major covariates, multivariate logistic regression analysis disclosed that Hhcy was independently associated with increased risk of poor outcome (OR = 1.760; 95% CI: 1.069–2.896) but was not associated with the risk of HT (OR = 1.017; 95% CI: 0.495–2.087). In addition, we found no significant interaction between Hhcy and hypertension on the clinical outcome (*p* = 0.513) or HT (*p* = 0.170) after thrombolysis.

**Conclusion:** We found that Hhcy was an independent risk factor for poor outcome, but not an independent risk factor for HT after thrombolysis in ischemic stroke patients. In addition, there was no significant interaction of Hhcy and hypertension on the clinical outcome or HT after thrombolysis.

## Introduction

Ischemic stroke accounts for 80–85% of all strokes and is one of the leading causes of mortality and disability in China (1). Intravenous thrombolysis with recombinant tissue plasminogen activator (rtPA) is an effective treatment for ischemic stroke, but it also increases the risk of hemorrhagic transformation (HT) (2–4). Symptomatic intracerebral hemorrhage (sICH) is the most severe hemorrhagic complication of thrombolytic therapy, which is associated with early neurological deterioration and worsened clinical outcome (4).

Hyperhomocysteinemia (Hhcy) is a condition in which the plasma levels of homocysteine and related metabolites are elevated. Accumulating evidence has demonstrated that Hhcy is an independent risk factor for ischemic stroke (5). However, the role of Hhcy in the clinical outcome of ischemic stroke remains controversial. Some studies found that Hhcy was associated with increased risk of poor outcome in ischemic stroke patients (6–11), whereas some studies showed no significant association between Hhcy and the clinical outcome of ischemic stroke (12–16). In addition, previous studies have found that Hhcy was implicated in the disruption of the blood-brain barrier, and the breakdown of the blood-brain barrier played a critical role in the development of HT in ischemic stroke patients with thrombolysis (17, 18). Thus, the aim of this study was to investigate the effect of Hhcy on the clinical outcome and HT after thrombolysis in patients with ischemic stroke.

## Materials and Methods

This study was approved by the Ethics Review Committee of the First Hospital of Jilin University, and written informed consent was obtained from all participants or their direct relatives.

### Participants

**Figure 1:**
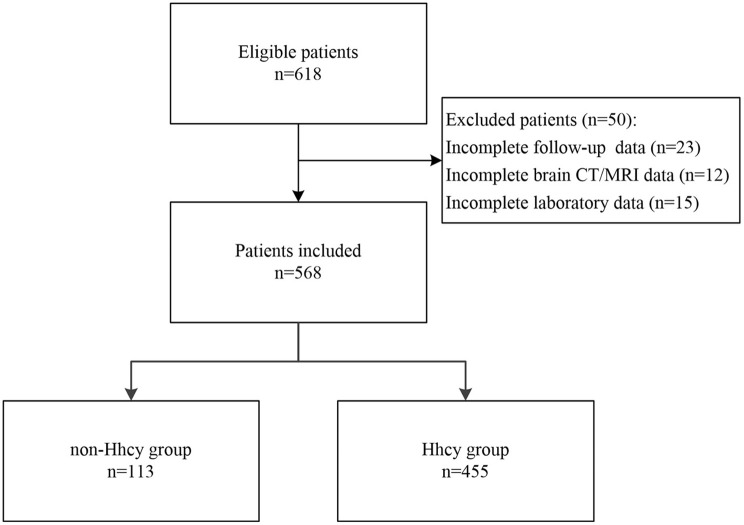
The flow chart of the study.

We consecutively recruited patients who were diagnosed with ischemic stroke and received intravenous thrombolysis with rtPA in our department between January 2016 and September 2018 for this study. The diagnosis of ischemic stroke was based on the clinical manifestations and signs, brain computed tomography (CT), and/or magnetic resonance imaging (MRI), as well as routine laboratory tests. Intravenous thrombolytic therapy was administrated within 4.5 h of onset for patients with ischemic stroke according to the recommendations of current guidelines and the decision of the treating physician (2, 3). After thrombolysis, all patients received standard medical treatment and general care in the comprehensive stroke center of our hospital. Patients who were lost on follow-up or missed data were excluded from this study. Patients were divided into Hhcy group and non-Hhcy group according to the levels of plasma homocysteine. A flow chart of the study is given in Figure 1.

### Data Collection

Demographics, vascular risk factors, previous history of drugs, as well as clinical and follow-up information of participants were prospectively collected and recorded in a well-established database. All information was obtained by trained staff through a standard questionnaire. Cigarette smoking was defined as having smoked at least 1 cigarette per day for 1 year or more (19). Alcohol consumption was defined as consuming 1 or more alcoholic drinks per day during the last year (19). Atrial fibrillation was defined as having a history of atrial fibrillation or a clinical diagnosis of atrial fibrillation during hospitalization (20). Coronary heart disease was defined as having a history of coronary heart disease or a clinical diagnosis of coronary heart disease during hospitalization. Hypertension was defined as having a history of hypertension, taking oral antihypertensive drugs, or having a clinical diagnosis of hypertension during hospitalization (20). Diabetes mellitus was defined as having a history of diabetes mellitus, taking oral hypoglycemic agents/insulin, or having a clinical diagnosis of diabetes mellitus during hospitalization (20). Dyslipidemia was defined as having a history of any types of dyslipidemia, taking oral antidyslipidemic drugs, or having at least one of the following findings during hospitalization: Total cholesterol ≥5.18 mmol/L, triglycerides ≥1.70 mmol/L, low-density lipoprotein cholesterol ≥3.37 mmol/L, and high-density lipoprotein cholesterol < 1.04 mmol/L (20, 21). Previous stroke was defined as having a history of transient ischemic attack, ischemic stroke, intracerebral hemorrhage, or subarachnoid hemorrhage (20). Previous history of drugs was defined as taking certain drugs regularly before admission. Plasma homocysteine levels were measured in the fasting state within 24 h after admission. Total homocysteine level ≥10 μmol/L was defined as Hhcy, which was recommended by the American Heart Association and the American Stroke Association Council on stroke (22, 23). Based on the dose of rtPA, intravenous thrombolysis was classified as standard-dose (0.9 mg/Kg) thrombolysis and low-dose (0.6 mg/Kg) thrombolysis. Bridging therapy was defined as receiving mechanical thrombectomy after intravenous thrombolysis. The characteristics of cerebral infarct were evaluated on the brain CT/MRI after thrombolysis. Infarct locations were divided into cortex, subcortex, thalamus, brainstem, and cerebellum, as previously reported (24). Infarct volume was measured on brain CT/diffusion-weighted MRI by ITK-SNAP 3.6.0 (25).

### Outcome

The clinical outcome was assessed at 3 months by modified Rankin Scale (mRS) with a score ranging from 0 (no symptoms) to 6 (death). We defined mRS scores ≥3 as a poor outcome and mRS scores ≤ 2 as a favorable outcome. HT was defined as any visible hemorrhage on brain CT or MRI 22–36 h after thrombolysis. sICH was defined as a HT that led to death or neurological deterioration with an increase of 4 or more on the National Institute of Health Stroke Scale (NIHSS) scores (26).

### Statistical Analysis

The data were analyzed by Statistical Program for Social Sciences version 22.0 (SPSS, IBM, West Grove, PA, USA). Continuous variables were expressed as means and standard deviation if the data was normally distributed, and vice versa by median and interquartile range. Categorical variables were expressed as frequency and percentage. The intergroup difference was compared using Student's *t*-test or Mann–Whitney test for continuous variables and the chi-squared test or Fisher's exact test for categorical variables. For exploring the effect of Hhcy on the clinical outcome and HT after thrombolysis, we first conducted univariate analysis to compare baseline factors associated with clinical outcome and HT. The variates with a *p*-value < 0.1 in the univariate analysis were included in multivariate logistic regression models as the major covariates. Then, we conducted multivariate logistic regression analysis to assess the association between Hhcy, clinical outcome, and HT after thrombolysis, and odd ratios (ORs) and 95% confidence intervals (95% CIs) were used to evaluate the risk of poor outcome and HT. Furthermore, we also assessed the potential interaction between Hhcy and hypertension on the clinical outcome and HT after thrombolysis. All statistical tests were two-tailed, and *p* < 0.05 was considered statistically significant.

## Results

### Overall Characteristics of Study Participants

A total of 618 patients were screened for this study, of which 23 patients were lost for follow-up, 12 patients missed the data of brain CT/MRI after thrombolysis, and 15 patients did not receive the examination of plasma homocysteine. Finally, 568 patients (418 men and 150 women) were included, and the overall characteristics of participants are shown in Table 1. The mean age was 62.2 ± 11.9 years. The median NIHSS score on admission was 9 (interquartile range, 5–13). The median time to treat was 3.0 h (interquartile range, 2.3–3.8 h). Previous history of drugs included antihypertensive drugs, hypoglycemic agents/insulin, statins, and anti-thrombotic agents. More than a quarter of participants received low-dose thrombolysis, and about 10% of patients underwent mechanical thrombectomy after intravenous thrombolysis. Of 568 patients, 455 (80.1%) had Hhcy, 66 (11.6%) had HT, 10 (1.8%) had sICH, and 219 (38.6%) had poor outcome.

**Table 1:** Patient characteristics between Hhcy group and non-Hhcy group.

**Variables**	**Total patients (*n* = 568)**	**non-Hhcy group (*n* = 113)**	**Hhcy group (*n* = 455)**	***p***
**Demographics**				
Age (year)	62.2 ± 11.9	58.7 ± 10.6	63.1 ± 12.1	0.001
Sex (male)	418 (73.6%)	63 (55.8%)	355 (78.0%)	0.001
**Vascular risk factors**				
Cigarette smoking	327 (57.6%)	53 (46.9%)	274 (60.2%)	0.010
Alcohol consumption	229 (40.3%)	45 (39.8%)	184 (40.4%)	0.905
Atrial fibrillation	100 (17.6%)	19 (16.8%)	81 (17.8%)	0.805
Coronary heartdisease	88 (15.5%)	17 (15.0%)	71 (15.6%)	0.883
Hypertension	338 (59.5%)	66 (58.4%)	272 (59.8%)	0.790
Diabetes mellitus	181 (31.9%)	50 (44.2%)	131 (28.8%)	0.002
Dyslipidemia	353 (62.1%)	75 (66.4%)	278 (61.1%)	0.301
Previous stroke	123 (21.7%)	26 (23.0%)	97 (21.3%)	0.696
**Previous history of drugs**				
Antihypertensive drugs	175 (30.8%)	26 (23.0%)	149 (32.7%)	0.045
Hypoglycemic agents/insulin	76 (13.4%)	24 (21.2%)	52 (11.4%)	0.006
Statins	5 (0.9%)	2 (1.8%)	3 (0.7%)	0.260
Anti-thrombotic agents	67 (11.8%)	15 (13.3%)	52 (11.4%)	0.586
**Baseline parameters**				
NIHSS score	9 (5-13)	(4-12)	9 (5-13)	0.334
SBP (mmHg)	157.8 ± 26.5	161.7 ± 27.9	156.9 ± 26.1	0.081
DBP (mmHg)	90 (80–100)	92 (82–100)	90 (79–100)	0.268
Blood glucose (mmol/L)	7.02 (6.11–8.72)	7.63 (6.45–10.94)	6.88 (6.00–8.48)	0.001
Homocysteine level (μmol/L)	13.5 (10.4–20.3)	8.6 (7.7–9.2)	14.8 (12.3–24.3)	0.001
**Treatment**				
Time to treat (hour)	3.0 (2.3–3.8)	3.1 (2.3–3.9)	3.0 (2.3–3.8)	0.321
Low-dose thrombolysis	157 (27.6%)	29 (25.7%)	128 (28.1%)	0.600
Bridging therapy	54 (9.5%)	8 (7.1%)	46 (10.1%)	0.326
**Characteristics of infarct**				
Cortex	214 (37.7%)	33 (29.2%)	181 (39.8%)	0.038
Subcortex	407 (71.7%)	81 (71.7%)	326 (71.6%)	0.994
Thalamus	63 (11.1%)	10 (8.8%)	53 (11.6%)	0.396
Brainstem	93 (16.4%)	24 (21.2%)	69 (15.2%)	0.118
Cerebellum	38 (6.7%)	7 (6.2%)	31 (6.8%)	0.814
Infarct volume (mL)	20.1 (10.0–40.1)	20.0 (9.8–39.6)	20.3 (10.1–40.3)	0.048
**Outcome**				
HT	66 (11.6%)	12 (10.6%)	54 (11.9%)	0.711
sICH	10 (1.8%)	1 (0.9%)	9 (2.0%)	0.695
Poor outcome	219 (38.6%)	33 (29.2%)	186 (40.9%)	0.022

*Hhcy, hyperhomocysteinemia; NIHSS, National Institute of Health Stroke Scale; SBP, systolic blood pressure; DBP, diastolic blood pressure; HT, hemorrhagic transformation; sICH, symptomatic intracerebral hemorrhage*.

### Patient Characteristics Between Hhcy Group and Non-Hhcy Group

The patient characteristics between Hhcy group and non-Hhcy group are compared in the Table 1. We found that patients with Hhcy were usually older and had a lower baseline blood glucose level than patients with non-Hhcy. In addition, patients with Hhcy had a higher proportion of male and cigarette smoking but a lower prevalence of diabetes mellitus than patients with non-Hhcy. Furthermore, Hhcy group had a higher proportion of antihypertensive treatment and a lower proportion of hypoglycemic treatment before admission compared with non-Hhcy group. For the specific types of drugs, there was no significantly statistical difference in the use of different types of antihypertensive drugs between the two groups. However, patients with non-Hhcy had a higher proportion of biguanides use than patients with Hhcy (Supplemental Table 1). Moreover, patients with Hhcy had a higher proportion of cortex infarct and a larger infarct volume than patients with non-Hhcy. As for the outcome, the Hhcy group had a higher incidence of poor outcome than the non-Hhcy group (40.9 vs. 29.2%, *p* = 0.022). However, there was no significant difference in the incidence of HT (11.9 vs. 10.6%, *p* = 0.711) and sICH (2.0 vs. 0.9%, *p* = 0.695) between the two groups.

### Association Between Hhcy and Clinical Outcome After Thrombolysis

**Figure 2:**
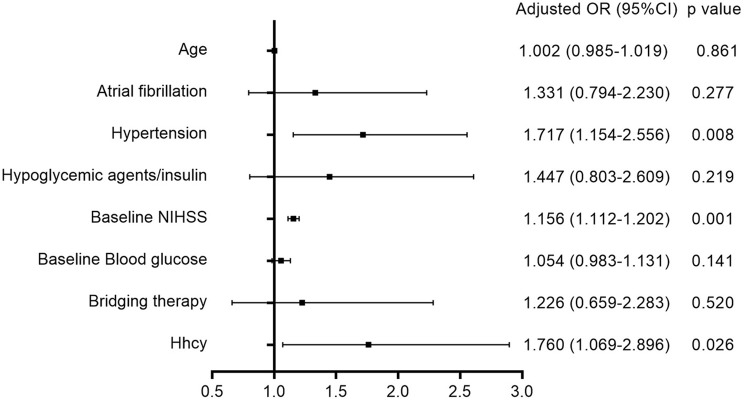
Association between Hhcy and poor outcome after thrombolysis.

As shown in Table 2, univariate analysis showed that poor outcome was associated with age, atrial fibrillation, hypertension, baseline NIHSS, baseline blood glucose, and bridging therapy. After adjustment for age, atrial fibrillation, hypertension, hypoglycemic agents/insulin, baseline NIHSS, baseline blood glucose, and bridging therapy, multivariate logistic regression analysis (Figure 2) disclosed that Hhcy was independently associated with an increased risk of poor outcome (OR = 1.760; 95% CI: 1.069–2.896). In addition, we found no significant interaction between Hhcy and hypertension on the clinical outcome after thrombolysis (*p* = 0.513).

**Table 2:** Univariate analysis of baseline factors associated with clinical outcome.

**Variables**	**Favorable outcome (*n* = 349)**	**Poor outcome (*n* = 219)**	***p***
**Demographics**			
Age (year)	61.1 ± 11.9	64.0 ± 11.8	0.005
Sex (male)	254 (72.8%)	164 (74.9%)	0.579
**Vascular risk factors**			
Cigarette smoking	207 (59.3%)	120 (54.8%)	0.289
Alcohol consumption	139 (39.8%)	90 (41.1%)	0.764
Atrial fibrillation	46 (13.2%)	54 (24.7%)	0.001
Coronary heart disease	55 (15.8%)	33 (15.1%)	0.825
Hypertension	196 (56.2%)	142 (64.8%)	0.040
Diabetes mellitus	103 (29.5%)	78 (35.6%)	0.129
Dyslipidemia	220 (63.0%)	133 (60.7%)	0.581
Previous stroke	76 (21.8%)	47 (21.5%)	0.929
**Previous history of drugs**			
Antihypertensive drugs	104 (29.8%)	71 (32.4%)	0.510
Hypoglycemic agents/insulin	40 (11.5%)	36 (16.4%)	0.090
Statins	4 (1.1%)	1 (0.5%)	0.654
Anti-thrombotic agents	37 (10.6%)	30 (13.7%)	0.265
**Baseline parameters**			
NIHSS score	7 (4–11)	12 (8–15)	0.001
SBP (mmHg)	156 (138–175)	160 (140–176)	0.603
DBP (mmHg)	90 (80–99)	91 (81–102)	0.259
Blood glucose (mmol/L)	6.78 (5.96–8.67)	7.26 (6.38–8.72)	0.022
Hhcy	269 (77.1%)	186 (84.9%)	0.022
**Treatment**			
Time to treat (hour)	3.0 (2.4–3.8)	3.0 (2.2–3.8)	0.623
Low-dose thrombolysis	97 (27.8%)	60 (27.4%)	0.918
Bridging therapy	27 (7.7%)	27 (12.3%)	0.069

*NIHSS, National Institute of Health Stroke Scale; SBP, systolic blood pressure; DBP, diastolic blood pressure; Hhcy, hyperhomocysteinemia*.

### Association Between Hhcy and HT After Thrombolysis

**Figure 3:**
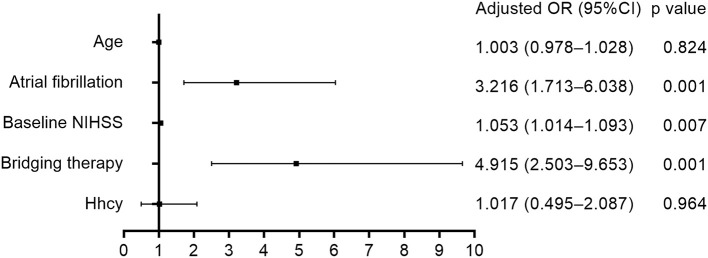
Association between Hhcy and HT after thrombolysis.

Table 3 shows that HT after thrombolysis was related to atrial fibrillation, baseline NIHSS, and bridging therapy. After adjustment for age, atrial fibrillation, baseline NIHSS, and bridging therapy, multivariate logistic regression analysis (Figure 3) disclosed that Hhcy was not associated with the risk of HT after thrombolysis (OR = 1.017; 95% CI: 0.495–2.087). In addition, we found no significant interaction between Hhcy and hypertension on the HT after thrombolysis (*p* = 0.170).

**Table 3:** Univariate analysis of baseline factors associated with HT.

**Variables**	**Patients without HT (*n* = 502)**	**Patients with HT (*n* = 66)**	***p***
**Demographics**			
Age (year)	61.9 ± 12.0	64.8 ± 10.9	0.062
Sex (male)	370 (73.7%)	48 (72.7%)	0.865
**Vascular risk factors**			
Cigarette smoking	290 (57.8%)	37 (56.1%)	0.792
Alcohol consumption	202 (40.2%)	27 (40.9%)	0.917
Atrial fibrillation	73 (14.5%)	27 (40.9%)	0.001
Coronary heart disease	75 (14.9%)	13 (19.7%)	0.315
Hypertension	302 (60.2%)	36 (54.5%)	0.382
Diabetes mellitus	161 (32.1%)	20 (30.3%)	0.772
Dyslipidemia	314 (62.5%)	39 (59.1%)	0.586
Previous stroke	106 (21.1%)	17 (25.8%)	0.389
**Previous history of drugs**			
Antihypertensive drugs	154 (30.7%)	21 (31.8%)	0.850
Hypoglycemic agents/insulin	70 (13.9%)	6 (9.1%)	0.276
Statins	5 (1.0%)	0 (0.0%)	1.000
Anti-thrombotic agents	56 (11.2%)	11 (16.7%)	0.192
**Baseline parameters**			
NIHSS score	8 (4–12)	12.5 (8–16)	0.001
SBP (mmHg)	157.7 ± 26.7	159.2 ± 25.2	0.660
DBP (mmHg)	90 (80–100)	91.5 (80–99)	0.764
Blood glucose (mmol/L)	7.01 (6.05–8.73)	7.24 (6.35–8.64)	0.398
Hhcy	401 (79.9%)	54 (81.8%)	0.711
**Treatment**			
Time to treat (hour)	3.0 (2.3–3.8)	2.8 (2.2–3.6)	0.444
Low-dose thrombolysis	142 (28.3%)	15 (22.7%)	0.342
Bridging therapy	35 (7.0%)	19 (28.8%)	0.001

*HT, hemorrhagic transformation; NIHSS, National Institute of Health Stroke Scale; SBP, systolic blood pressure; DBP, diastolic blood pressure; Hhcy, hyperhomocysteinemia*.

## Discussion

This study included a cohort of ischemic stroke patients with intravenous rtPA thrombolysis and attempted to determine the effect of Hhcy on the clinical outcome and HT after thrombolysis. We found that Hhcy was an independent risk factor for poor outcome, but not an independent risk factor for HT after thrombolysis. In addition, we found no significant interaction of Hhcy and hypertension on the clinical outcome or HT after thrombolysis.

Although a small number of studies have already been published, the role of Hhcy in the clinical outcome of ischemic stroke after thrombolysis remains controversial. Ribo et al. first explored this issue in two prospective studies of small sample sizes (44 patients and 77 patients, respectively) and found that plasma homocysteine levels were not associated with the clinical outcome after thrombolysis in ischemic stroke patients (15, 16). However, in a retrospective study of 194 patients with ischemic stroke, Yao and colleagues found that compared with the first quartile (2.26–11.50 μmol/L) of serum homocysteine levels, the fourth quartile (23.85–138.90 μmol/L) had the highest risk of poor outcome after thrombolysis (OR = 13.65; 95% CI: 3.58–51.97) (10). Another retrospective study included 120 ischemic stroke patients and also found that compared with the first quartile (2.26–11.50 μmol/L) of serum homocysteine levels, the fourth quartile (23.85–138.90 μmol/L) was independently associated with an increased risk of poor outcome after thrombolysis (OR = 13.75; 95% CI: 3.57–51.77) (11). These inconsistent results might be caused by small sample sizes. Thus, we conducted this study with a larger sample size and adopted the definition of Hhcy recommended by the American Heart Association and the American Stroke Association Council on stroke to investigate the effect of Hhcy on the clinical outcome after thrombolysis. We found that consistent with the findings of the two retrospective studies described above, Hhcy was an independent risk factor for poor outcome, which provides new proof for the adverse effects of Hhcy on the clinical outcome of ischemic stroke.

The mechanisms of Hhcy's effect on the clinical outcome of ischemic stroke after thrombolysis are not clear. However, experimental studies have provided some insights into the mechanisms of Hhcy-induced brain injury. It was found that Hhcy can induce excitotoxicity via the glutamate receptor (27). In addition, Hhcy can increase the production of free radicals and inhibit the activity of antioxidant enzymes to cause oxidative stress (27). Furthermore, Hhcy can increase the expression of pro-inflammatory genes in microglia to mediate an inflammatory response (28). Moreover, Hhcy can lead to neuronal death via multiple mechanisms: Hhcy can induce DNA damage, mitochondrial dysfunction, and the stress response of endoplasmic reticulum to cause neural cell apoptosis (29); Hhcy can lead to neuronal death via oxidative damage-mediated autophagy activation (30); Autoxidation of Hhcy metabolites leads to the production of large amounts of H_2_0_2_ that can induce necrotic cell death (27).

Previous research has found that HT after thrombolysis was associated with rtPA-induced coagulopathy, ischemic injury, reperfusion injury, and disruption of the blood-brain barrier, and the breakdown of blood-brain barrier played a critical role in the development of HT (4, 17, 31). Experimental studies found that Hhcy can lead to endothelial dysfunction, increased expression of matrix metalloproteinase 9, and disruption of the blood-brain barrier (18, 27, 28). Furthermore, a clinical study found that higher serum homocysteine levels were independently associated with an elevated risk of cerebral microbleeds in patients with acute ischemic stroke due to large-artery atherosclerosis (32). These findings imply that Hhcy might increase the risk of HT in ischemic stroke patients with intravenous thrombolysis. However, we did not find a significant association between Hhcy and HT after thrombolysis in this study. To our best knowledge, it is the first study to explore the relationship between Hhcy and HT after thrombolysis. In addition, there was no significantly statistical difference in the incidence of sICH between Hhcy group and non-Hhcy group in the present study. However, previous studies found that patients with Hhcy tended to have a higher incidence of sICH (10, 11). Thus, the association between Hhcy and sICH needs further investigation in the future.

In China, Hhcy is a common comorbidity in hypertensive patients with a proportion of up to 75% (19). Previous studies have noted an interaction between Hhcy and hypertension on some clinical events. For example, a case-control study found a more than multiplicative effect of Hhcy and hypertension on the risk of vascular disease (33). In addition, Fan and colleagues also found that Hhcy and hypertension had a more than multiplicative effect on the baseline stroke severity in patients with first-ever ischemic stroke (34). However, this effect has not been assessed on the clinical outcome or HT in ischemic stroke patients with intravenous thrombolysis. In this study, we did not find significant interaction between Hhcy and hypertension on the clinical outcome or HT after thrombolysis in ischemic stroke patients.

There are some limitations in the present study. First, this study was a retrospective analysis with a limited sample size. Second, the levels of plasma homocysteine were measured after thrombolysis, which might affect the correlation between Hhcy and HT. Third, Hhcy may be caused by genetic factors, physiologic factors, lifestyle factors, various diseases, and drugs (35). However, we did not assess the cause of Hhcy in this study cohort, such as genetic testing. Last, we did not explore the relationship between Hhcy and sICH. Further exploration should be conducted in a multicenter study with a large sample size.

## Conclusion

We found that Hhcy was an independent risk factor for poor outcome, but not an independent risk factor for HT after thrombolysis in ischemic stroke patients. In addition, there was no significant interaction of Hhcy and hypertension on the clinical outcome or HT after thrombolysis.

## Data Availability

The datasets generated for this study are available on request to the corresponding author.

## Ethics Statement

This study was approved by the Ethics Review Committee of the First Hospital of Jilin University, and written informed consent was obtained from all participants or their direct relatives.

## Author Contributions

YL, XS, and YiY were responsible for study design. HJ, Z-NG, L-YZ, JC, YaY, YW, JL, and Q-YH performed the data collection. PZ conducted the data analysis. YL drafted the manuscript. HJ, Z-NG, and PZ helped revise the article. All authors were responsible for data interpretation.

### Conflict of Interest Statement

The authors declare that the research was conducted in the absence of any commercial or financial relationships that could be construed as a potential conflict of interest.
