# Supplementary material for: Effect of Hyperhomocysteinemia on Clinical Outcome and Hemorrhagic Transformation After Thrombolysis in Ischemic Stroke Patients
Source: Front Neurol. 2019 Jun 4;10:592. doi: 10.3389/fneur.2019.00592 (PMC6584786; doi:10.3389/fneur.2019.00592)
Supplement: Supplementary file 1 [file Table_1.DOCX]

**Supplemental Table 1** Comparison of previous history of drugs between patients with Hhcy and non-Hhcy

| **Variables** | | **non-Hhcy group**  **(n=113)** | **Hhcy group**  **(n=455)** | **P** |
| --- | --- | --- | --- | --- |
| **Antihypertensive drugs** | | 26 (23.0%) | 149 (32.7%) | 0.045 |
|  | Diuretics | 2 (1.8%) | 1 (0.2%) | 0.102 |
|  | Calcium channel blockers | 26 (23.0%) | 142 (31.2%) | 0.087 |
|  | Beta-blockers | 0 (0.0%) | 3 (0.7%) | 1.000 |
|  | ACE/angiotensin II receptor inhibitors | 0 (0.0%) | 3 (0.7%) | 1.000 |
| **Hypoglycemic agents/insulin** | | 24 (21.2%) | 52 (11.4%) | 0.006 |
|  | Sulfonylurea | 1 (0.9%) | 0 (0.0%) | 0.199 |
|  | Biguanides | 7 (6.2%) | 2 (0.4%) | 0.001 |
|  | Glinides | 1 (0.9%) | 1 (0.2%) | 0.359 |
|  | Insulin | 16 (14.2%) | 50 (11.0%) | 0.347 |
| **Statins** | | 2 (1.8%) | 3 (0.7%) | 0.260 |
| **Anti-thrombotic agents** | | 15 (13.3%) | 52 (11.4%) | 0.586 |
|  | Anticoagulants | 1 (0.9%) | 3 (0.7%) | 1.000 |
|  | Anti-platelet agents | 15 (13.3%) | 50 (11.0%) | 0.495 |

ACE: angiotensin converting enzyme.
